# Effectiveness of Adding Metacognitive Training to Occupational Therapy in Patients With Schizophrenia Under Long-Term Hospitalization: A Pilot Randomized Controlled Trial

**DOI:** 10.7759/cureus.88476

**Published:** 2025-07-21

**Authors:** Rumi Sunohara, Shohei Ichikawa, Daiki Saito, Ai Tayama, Mizuki Nakajima, Masayoshi Kobayashi

**Affiliations:** 1 Department of Health Sciences, Graduate School of Medicine, Shinshu University, Nagano, JPN; 2 Department of Occupational Therapy, Medical Corporation Uematsu Hospital, Nagano, JPN; 3 Department of Health Sciences, Graduate School of Medicine, Shinshu University, Matsumoto, JPN

**Keywords:** beck cognitive insight scale, elderly, global assessment of functioning, long-term hospitalization, metacognitive training, montreal cognitive assessment (moca), occupational therapy, panss, randomized controlled trial, schizophrenia

## Abstract

Objective

This study investigated the effects of metacognitive training (MCT) on improving psychiatric symptoms and cognitive function in patients with schizophrenia who were hospitalized for long periods.

Methods

The participants were long-term inpatients with schizophrenia, hospitalized in a private psychiatric hospital in Japan. Participants were randomly assigned to either the occupational therapy (OT)+MCT group or the OT-alone group. The OT+MCT group received 16 weekly MCT sessions, each lasting 60 minutes, over a period of four months. The Japanese versions of the Montreal Cognitive Assessment (MoCA-J), Positive and Negative Syndrome Scale (PANSS), Beck Cognitive Insight Scale (BCIS), and Global Assessment of Functioning (GAF) scores were compared before and after the intervention using a two-way repeated measures analysis of variance. To examine age-related effects, a correlation analysis was performed between participants’ age and their MoCA-J total score. Furthermore, the OT+MCT group was stratified by median age (68 years), and changes in each outcome measure were compared between the groups.

Results

The 41 participants had a mean age of 69.22 years, ranging from 37 to 79 years, with 21 assigned to the OT+MCT group and 20 to the OT-alone group. There were no dropouts during the study period. The mean MoCA-J Total score at baseline was 17.67 (SD = 5.37) in the OT+MCT group and 14.85 (SD = 5.95) in the OT-alone group, indicating mild cognitive impairment in both groups. Four months after the intervention, both groups demonstrated an upward trend in MoCA-J scores. Notable improvements were seen in visuospatial/executive and total scores over time in both groups. However, no significant between-subject effects or interaction effects were detected. Additionally, a significant negative correlation was observed between age and the MoCA-J total score. In the PANSS, scores decreased in both groups at the post-intervention assessment, and significant differences were observed in general psychopathology and total score categories due to within-subjects factors. Negative symptom scores significantly differed between groups; however, no interaction with time was observed. Participants aged 68 or older showed greater reductions in PANSS scores. While no significant differences were observed in positive symptoms, significant improvements were observed in negative symptoms, general psychopathology, and overall PANSS scores. No significant differences were observed between the two groups in either the BCIS or GAF scores.

Conclusions

These findings suggest that MCT is feasible for long-term hospitalized elderly patients with schizophrenia and may improve psychiatric symptoms when combined with OT. Although OT+MCT did not significantly improve cognitive function, the results suggest it may be particularly beneficial for older patients in managing persistent psychiatric symptoms. Future studies should investigate the cognitive effects of MCT in larger samples, determine optimal treatment duration and frequency, and explore potential interactions with medications.

## Introduction

Mental health care in Japan faces significant challenges, including high suicide rates, a large number of psychiatric beds, and prolonged hospital stays [1]. Approximately 302,000 patients are currently hospitalized in psychiatric wards in Japan, of whom 154,000 (51%) have been diagnosed with schizophrenia [2]. Among these, approximately 200,000 patients have been institutionalized for over a year; of this group, 63.5% have schizophrenia, and 50% are aged 65 years or older [3].

A web-based survey of Japanese psychiatrists identified residual positive symptoms (46.5%) and cognitive decline (37.5%) as the primary reasons for continued hospitalization among long-term inpatients. The study emphasized that addressing these issues is essential to facilitate discharge [4]. Cognitive impairment in long-term hospitalized patients with schizophrenia can lead to significant declines in psychosocial functioning and daily living activities, underscoring the need for targeted interventions [5,6]. Furthermore, cognitive function in psychotic disorders has been reported to decline over an extended period, even exceeding that observed in normal aging [7]. Because thinking skills affect recovery so much, treatments that help them are needed [8,9].

Cognitive impairments in schizophrenia encompass both neurocognitive deficits, such as attention, memory, working memory, fluency, executive function, and processing speed, and cognitive biases, including deficits in theory of mind, causal attribution bias, and leap-to-conclusion. These cognitive biases contribute to delusions, false beliefs, and negative symptoms and affect daily functioning in long-term hospitalized patients [10,11]. Due to the limitations of pharmacotherapy for cognitive impairments in schizophrenia [12], several psychosocial treatment programs have been developed. These include (i) Neuropsychological Educational Approach to Cognitive Remediation (NEAR) [13], which focuses on neurocognitive training, (ii) the RehaCom® higher brain function training system [14], and (iii) metacognitive training (MCT) aimed at improving cognitive biases [15]. All of these approaches have shown promising results [8,9,15].

NEAR and RehaCom®, which require computer proficiency, may be unsuitable for programs targeting long-term patients with schizophrenia. A significant proportion of these patients are elderly and unfamiliar with digital technology, often leading to reluctance or avoidance of participation. In contrast, MCT is relatively easy to implement and encourages participation among elderly patients [16]. In MCT sessions, PowerPoint materials (Microsoft Corporation, Redmond, Washington, United States) called modules are presented, and participants practice recognizing cognitive biases through group discussions. These modules incorporate animations and interactive quizzes, making the sessions engaging while promoting cognitive flexibility and adaptive behaviors.

Previous studies have reported that MCT reduces positive symptoms in patients with schizophrenia [17], with improvements sustained for three years later [18]. The Japanese version of the MCT was developed in 2012 [19], and randomized controlled trials in Japan have reported improvements in positive symptoms, particularly delusions, as well as in overall functioning and cognitive biases in patients with schizophrenia [20]. However, limited evidence exists regarding the effectiveness of MCT in long-term hospitalized patients with schizophrenia. We aimed to investigate whether MCT is effective in improving psychiatric symptoms and cognitive function in long-term hospitalized patients with schizophrenia. Demonstrating MCT’s efficacy could inform future rehabilitation programs for chronically hospitalized patients with schizophrenia.

## Materials and methods

This study was conducted at the Uematsu Hospital, located in Nagano Prefecture, Japan, between November 2023 and March 2024. The study was approved by the Ethics Committee of the Faculty of Medicine, Shinshu University (approval number: 5181) and the Ethics Committee of Uematsu Hospital, and it was registered in the University Hospital Medical Information Network Clinical Trials Registry (UMIN-CTR) (registration number: UMIN000044956). All participants were informed of the study’s aim both in writing and verbally, and their consent was obtained.

Eligibility criteria

Inclusion criteria were as follows: Patients who had been voluntarily admitted to a psychiatric hospital for more than one year, diagnosed with schizophrenia according to the DSM-5 (American Psychiatric Association, 2013) diagnostic criteria by a psychiatrist, and receiving occupational therapy (OT) in a psychiatric setting. Exclusion criteria included diagnoses of dementia, epilepsy, alcohol or drug dependence, head injury, cerebrovascular disease, or unstable medical conditions that made it difficult to perform the MCT or cognitive function tests. Participants who were prone to interpersonal problems and were deemed ineligible by the attending physician or principal investigator were also excluded. The study population consisted of patients with schizophrenia who met all the inclusion criteria and did not meet any exclusion criteria. 

Randomization and intervention

Baseline assessments were conducted by independent research staff who had no contact with the patients who agreed to participate in the study. Participants who underwent baseline assessment were randomly assigned to either the OT+MCT group, which received OT and MCT, or the OT-alone group, which received only OT, using a computer-generated randomization program. Randomization was stratified by age (under 60, 61-70, and 71 or older) and sex (male/female), and participants were randomly assigned to the OT+MCT group and the OT alone group at a 1:1 ratio.

The OT+MCT group received a standard OT program along with 16 MCT sessions over a period of four months. The OT-alone group received only the standard OT program. Post-intervention evaluations were conducted after completion of the MCT program (four months after the baseline evaluation). The OT+MCT group included patients who participated in at least 10 of the 16 MCT sessions (62.5% of the sessions). The intervention was non-blinded; however, the evaluators were blinded to the treatment assignments.

The Japanese version of MCT [19], consisting of eight modules, was used in this study. The MCT program had 16 sessions across two rounds. Topics included how people think and make decisions. Participants in the OT+MCT group underwent 16 weekly MCT sessions, each lasting 60 minutes. Sessions were conducted in a fixed group of approximately 10 participants, led by two occupational therapists. PowerPoint materials were presented using a projector to promote interaction and engagement with the learning tasks. In addition, in regular OT, individual or group programs lasting 1.5-2 hours per session were provided four to five times a week, in accordance with the treatment goals of each participant. The regular OT program included physical exercise, constructive crafts, recreation, and psychoeducation. There was no difference in the number of individual and group program sessions between the OT+MCT group and the OT alone group. The total duration of the program was equivalent between the OT+MCT and OT-alone groups. All the patients received standard treatment during the study, including regular consultations with a psychiatrist, antipsychotic medications, and individual case management.

Measurements and tools

Demographic information included age, sex, number of hospitalizations, illness duration, education duration, current hospitalization duration (years), cumulative duration of hospitalization (years), employment experience, and antipsychotic medication dosage. Efficacy outcomes were collected at baseline and at four months. The primary efficacy endpoints were changes in psychiatric symptoms. Secondary efficacy outcomes included changes in cognitive function and cognitive insight, and functional levels.

The Japanese version of the Positive and Negative Syndrome Scale (PANSS) was used to assess psychiatric symptoms. This was purchased by the authors for use in this study. The PANSS is a 30-item rating scale designed to assess the severity of psychotic symptoms, including seven items for positive symptoms, seven items for negative symptoms, and 16 items for general psychopathology [21]. All items are rated on a scale from 1 (no symptoms) to 7 (extremely severe symptoms), with higher scores indicating greater symptom severity.

The Japanese version of the Montreal Cognitive Assessment (MoCA-J) [22,23] was used to evaluate cognitive function. Although originally developed to detect mild cognitive impairment (MCI), the MoCA is also effective in identifying cognitive deficits in schizophrenia. The MoCA has a significant correlation (r =.61, p <.001) with the Brief Assessment of Cognition in Schizophrenia (BACS), a tool for evaluating cognitive dysfunction in schizophrenia. In addition, it has high sensitivity in detecting cognitive impairments in these populations [24]. MoCA-J assesses the following domains: (i) visuospatial/executive, (ii) naming, (iii) attention, (iv) language, (v) abstraction, (vi) delayed recall, and (vii) orientation. The total score ranges from 0 to 30 points, and a cutoff point of 25/26 [23] is used to assess cognitive function. The MoCA-J was used in this study with permission from MoCA Cognition (https://mocacognition.com/).

Cognitive insight was evaluated using the Japanese version of the Beck Cognitive Insight Scale (BCIS) [25]. This is a 15-statement self-report measure that assesses cognitive biases. Participants were instructed to rate their agreement with each statement on a 4-point scale. Scores were calculated by summing the nine items of Self-reflectiveness (SR) and the six items of Self-certainty (SC), with the Composite index (range: -18 to 27) derived by subtracting SR from SC. A higher composite index indicates greater cognitive insight. The Japanese version of the BCIS used in this study has demonstrated good validity, reliability, and internal consistency (Cronbach's α = 0.67-0.78) [25].

The Global Assessment of Functioning (GAF) [26] was used for the overall functional assessment. In Japan, the Ministry of Health, Labour and Welfare recommends the use of GAF, and its use is mandatory for assessments in psychiatric home care. The GAF is a subjective measure that evaluates an individual's social, occupational, and psychological functioning (ability to cope with life problems), with scores ranging from 100 (extremely high functioning) to 1 (severe functional impairment).

Assessments were conducted by different professionals: the PANSS was assessed by psychiatrists, while the MoCA-J, BCIS, and GAF were assessed by occupational therapists.

Statistical analysis

A protocol-specific analysis was performed. Basic demographic variables were compared between groups using t-tests and/or chi-square tests. Analysis of evaluation scores (i.e., scores on evaluation scales) was performed using a two-way repeated-measures analysis of variance (ANOVA), with Time (baseline vs. post-intervention) as the within-subjects factor and Group (OT+MCT vs. OT only) as the between-subjects factor. Furthermore, to investigate age-related effects, a correlation analysis was performed between participants' age and cumulative hospitalization period and the total MoCA-J score. Additionally, the OT+MCT group was stratified by age (median: 68 years), and changes in each outcome measure were compared between groups. Statistical analyses were conducted using Bell Curve Excel Statistics version. 4.09 (Social Survey Research Information Co., Ltd., Tokyo, Japan), compatible with Microsoft Excel (Microsoft Corporation). The significance level was set at p <.05 for both sides.

## Results

**Figure 1:**
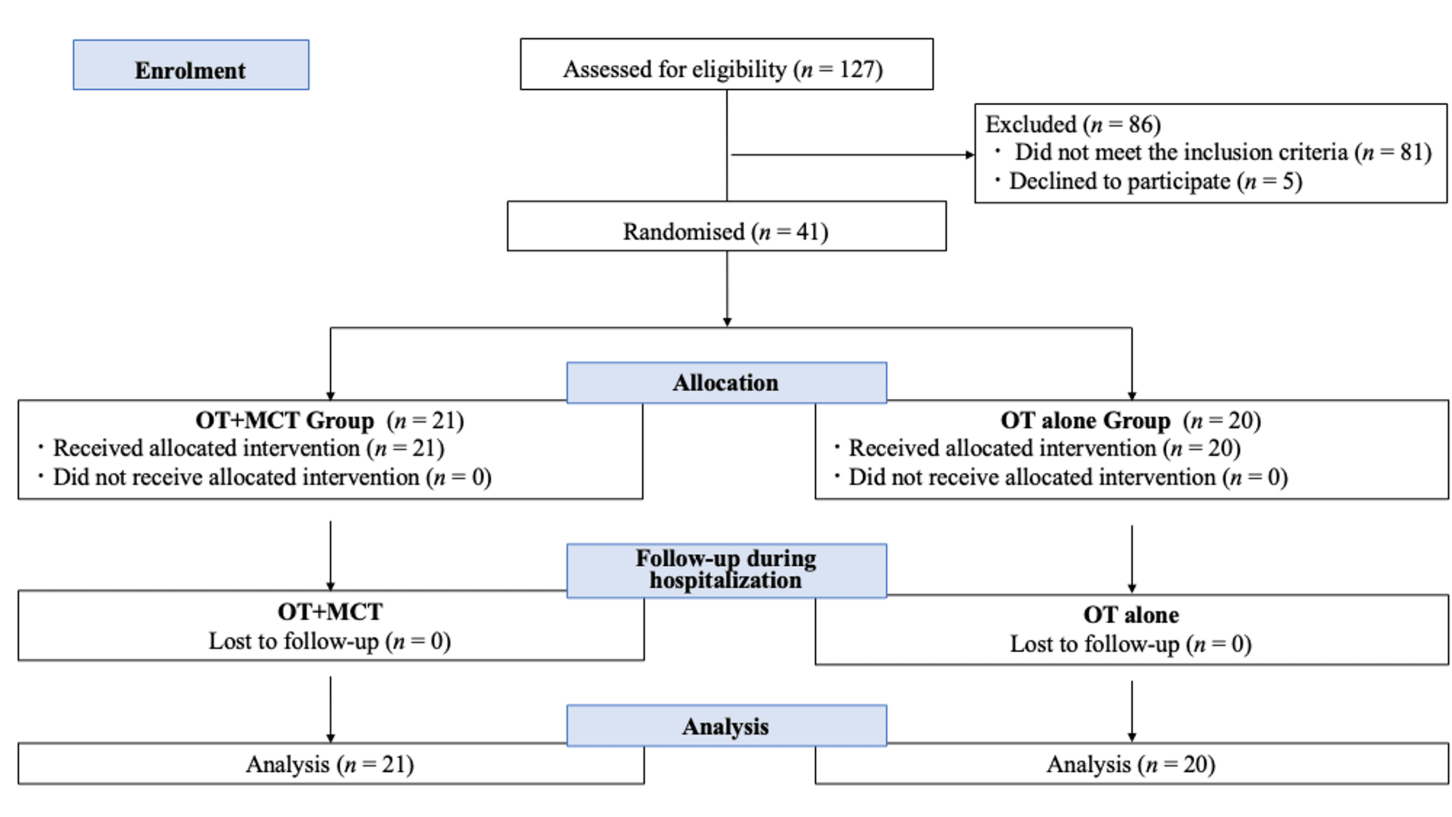
Study flow chart OT, occupational therapy; MCT, metacognitive training

A flowchart of the study process is shown in Figure 1. Of the 127 patients hospitalized in psychiatric wards screened for eligibility, 41 fulfilled the inclusion/exclusion criteria and were enrolled. The mean age of the 41 randomized patients was 69.22 years (SD 8.67), with 26 male participants (63.41%). Of these 41, 21 patients were assigned to the OT+MCT group and 20 to the OT-alone group. There were no dropouts during the study period in either group, and the MCT participation rate was 94.2%, with all participants included in the analysis.

The demographic characteristics of the participants are shown in Table 1. The mean age was 67.19 (SD 10.73) years in the OT+MCT group and 71.35 (SD 5.40) years in the OT-alone group. The cumulative duration of hospitalization was 28.60 years (SD 20.80) in the OT+MCT group and 23.80 years (SD 17.50) in the OT-alone group, indicating that both groups consisted mainly of elderly patients with schizophrenia who have been hospitalized for an extended period. There were no significant differences between the two groups in terms of age, sex distribution, illness duration, number of hospitalizations, years of education, current hospitalization duration, cumulative hospitalization duration, employment history, or antipsychotic medication dosage.

**Table 1:** Demographic characteristics of the participants ^a^ Chlorpromazine equivalent dose Group comparisons were performed using the Student’s t-test for continuous variables and the chi-square analyses for categorical variables. *p < 0.05, **p < 0.01. Data presented as mean±SD except for sex (male) and employment experience (yes), which are presented as frequency (n). OT, occupational therapy; MCT, metacognitive training

Variable	OT + MCT (n = 21)	OT Alone (n = 20)	t /χ2	p
	Mean ± SD	Mean ± SD		
Age (years)	67.19 ± 10.73	71.35 ± 5.40	1.55	0.13
Sex (male), n	13	13	0.04	0.84
Duration of illness (years)	44.10 ± 13.95	43.45 ± 11.86	0.16	0.87
Number of hospitalizations	4.33 ± 2.87	5.30 ± 5.05	0.76	0.45
Education (years)	12.19 ± 2.06	11.75 ± 1.74	0.74	0.46
Current hospitalization duration (years)	10.66 ± 10.47	8.52 ± 9.92	0.81	0.42
Cumulative duration of hospitalization (years)	28.60 ± 20.80	23.80 ± 17.50	1.03	0.31
Employment experience (yes), n	17	19	1.89	0.17
Antipsychotic dosage (mg/day)^a^				
Baseline	635.91 ± 467.25	501.10 ± 430.94	0.96	0.40
Post-intervention (four months)	645.43 ± 464.23	499.85 ± 431.98	1.04	0.31

Changes in the assessment scores

Table 2 presents the changes in assessment scores between the OT+MCT and OT-alone groups, as well as the results of a two-way repeated measures analysis of variance.

**Table 2:** Assessment score analysis by two-way repeated-measures ANOVA Two-way repeated-measures analysis of variance: *p < 0.05, **p < 0.01. Factors: Time (within-subjects factor), Group (between-subjects factor), and Time×Group (interaction) ES, Effect size (*η*^2^) for the Time×Group interaction: *η*^2^ = 0.01 (small), *η*^2 ^= 0.06 (medium), *η*^2^ = 0.14 (large). PANSS, Positive and Negative Syndrome Scale; MoCA-J, Japanese version of the Montreal Cognitive Assessment; BCIS, Beck Cognitive Insight Scale; GAF, Global Assessment of Functioning.

Measure	Time	OT + MCT (n = 21)	OT-alone (n = 20)	F			ES (η^2^)
		Mean ± SD	Mean ± SD	Time	Group	Time×Group	
PANSS							
･Positive	Baseline	20.57 ± 5.31	17.25 ± 4.14	1.99	3.50	0.89	0.00
	Post	19.05 ± 6.42	16.95 ± 3.94				
･Negative	Baseline	23.00 ± 5.56	26.85 ± 5.41	3.73	7.71**	2.97	0.01
	Post	21.24 ± 5.74	26.75 ± 5.72				
･General psychopathology	Baseline	45.29 ± 9.16	46.40 ± 6.75	12.21**	1.45	4.08	0.01
	Post	40.24 ± 9.76	45.05 ± 7.48				
･Total score	Baseline	88.86 ±17.09	90.50 ±11.98	9.02**	1.12	3.85	0.01
	Post	80.52 ±19.79	88.75 ±13.04				
MoCA-J							
･Visuospatial/Executive	Baseline	1.95 ± 1.40	1.45 ± 1.47	6.09*	1.86	0.19	0.00
	Post	2.52 ± 1.54	1.85 ± 1.66				
･Naming	Baseline	2.71 ± 0.56	2.30 ± 0.92	3.84	1.13	6.65*	0.02
	Post	2.67 ± 0.58	2.65 ± 0.67				
･Attention	Baseline	3.71 ± 1.74	3.20 ± 1.82	3.80	2.70	3.80	0.01
	Post	4.38 ± 1.69	3.20 ± 1.70				
･Language	Baseline	1.29 ± 0.90	0.75 ± 0.85	0.00	2.34	2.30	0.01
	Post	1.10 ± 0.70	0.95 ± 0.83				
･Abstraction	Baseline	0.81 ± 0.75	0.90 ± 0.85	0.06	0.34	3.37	0.02
	Post	1.00 ± 0.89	0.65 ± 0.75				
･Delayed Recall	Baseline	1.19 ± 1.63	0.80 ± 1.36	1.86	0.52	0.07	0.00
	Post	1.43 ± 1.86	1.15 ± 1.66				
･Orientation	Baseline	5.29 ± 1.01	4.70 ± 1.59	0.07	1.57	0.30	0.00
	Post	5.14 ± 1.28	4.75 ± 1.55				
･Total score	Baseline	17.67 ± 5.37	14.85 ± 5.95	11.15**	2.74	0.03	0.00
	Post	19.00 ± 5.18	16.05 ± 6.30				
BCIS							
･Self-reflectiveness	Baseline	10.52 ± 3.93	9.70 ± 5.03	0.81	1.93	1.36	0.01
	Post	12.10 ± 4.89	9.50 ± 4.62				
･Self-certainty	Baseline	7.85 ± 3.84	5.85 ± 3.50	4.56*	3.09	0.18	0.00
	Post	8.95 ± 3.56	7.50 ± 4.12				
･Composite index	Baseline	2.68 ± 5.04	3.85 ± 3.70	0.93	0.00	2.59	0.02
	Post	3.14 ± 6.00	2.00 ± 3.88				
GAF							
	Baseline	46.33 ±17.07	46.45 ±12.22	0.16	0.08	1.60	0.00
	Post	48.14 ±18.35	45.50 ±10.44				

**Figure 2:**
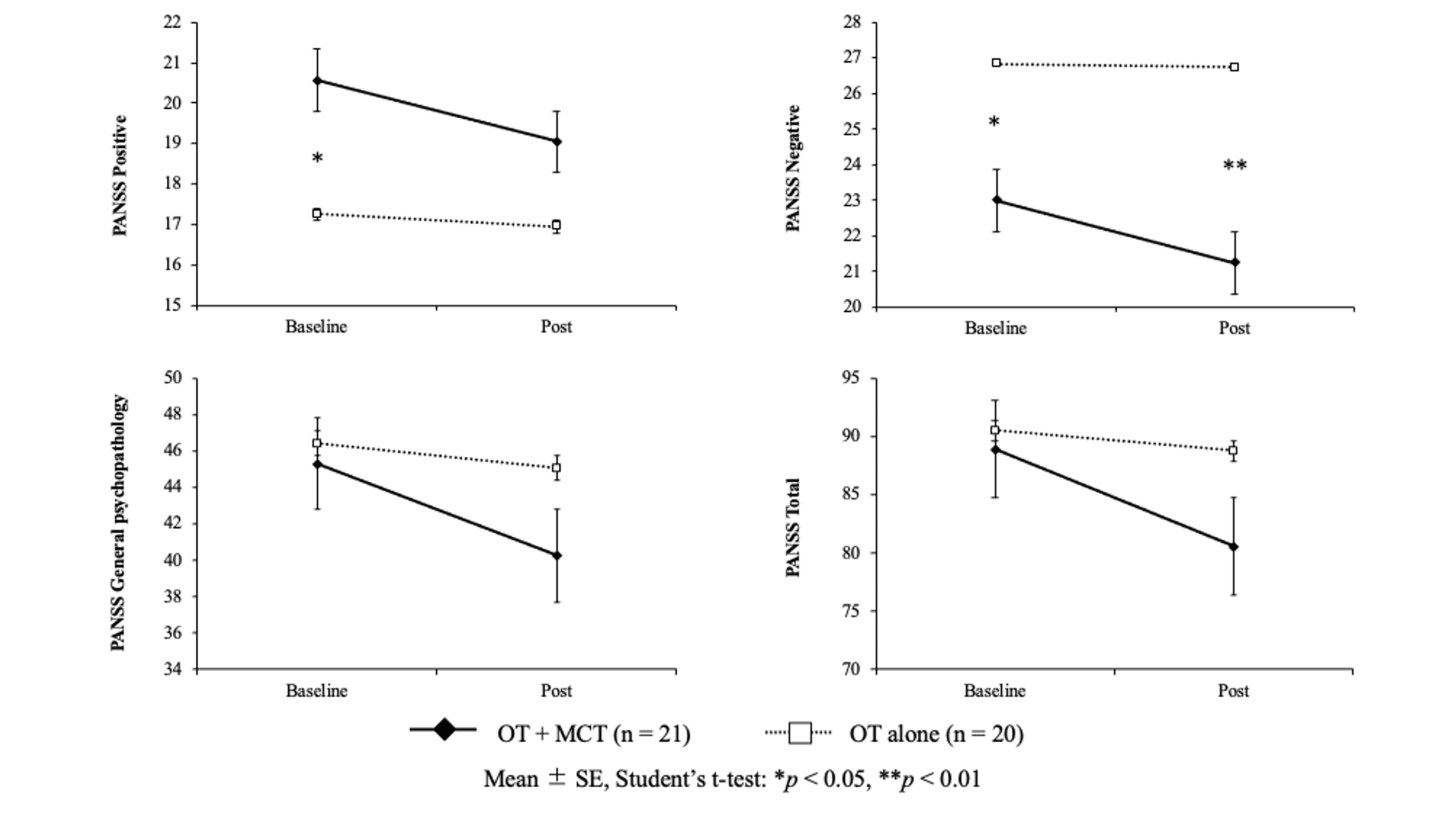
Change in PANSS subscale and total scores between baseline and at the end of the intervention after four months PANSS, Positive and Negative Syndrome Scale; OT, occupational therapy; MCT, metacognitive training

In the PANSS, both groups showed decreased scores in the post-intervention evaluation. Significant within-subjects effects were observed for general psychopathology (F = 12.21, p < 0.01, η2 = 0.04) and total score (F = 9.02, p < 0.01, η2 = 0.03). Additionally, negative symptoms showed a significant between-subjects effect (F = 7.71, p < 0.01, η² = 0.15), but no significant interaction with the within-subjects factor was observed (F = 2.97, p = 0.09, η² = 0.01). Changes in positive and negative symptoms, general psychopathology, and total PANSS scores are shown in Figure 2.

At baseline, the mean MoCA-J total score was 17.67 (SD = 5.37) in the OT+MCT group and 14.85 (SD = 5.95) in the OT-alone group, both of which were significantly below the cutoff value of 26/25. At the end of the intervention after four months, both groups showed an increasing trend in the MoCA-J Total Score and subscale scores. Significant within-subjects effects were found for the Visuospatial/Executive (F = 6.09, p = 0.02, η² = 0.03) subscale and the Total score (F = 11.15, p < 0.01, η² = 0.01), but no significant between-subjects effects were observed. Additionally, an interaction between the within-subjects factor and the between-subjects factor was observed in the Naming task (F = 6.65, p = 0.01, η2 = 0.02).

In the post-intervention evaluation of the BCIS, SC increased in both groups, with a significant within-subjects effect (F = 4.56, p = 0.04, η² = 0.03), but no interaction between the within-subjects and between-subjects factors was observed. Additionally, while not statistically significant, the BCIS Composite Index score increased in the OT+MCT group and decreased in the OT-alone group.

The influence of age 

**Figure 3:**
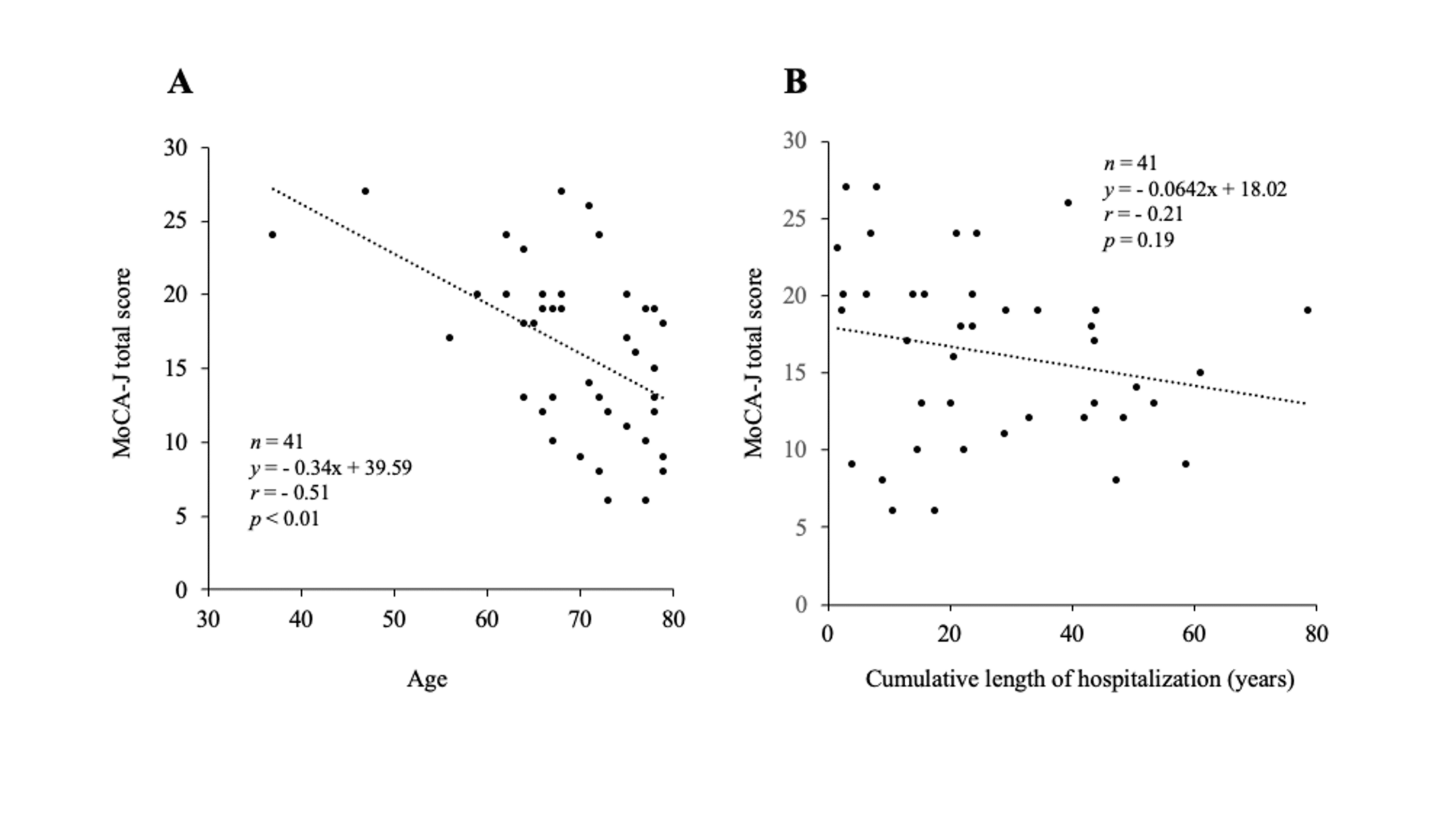
Correlation between (A) age and MoCA-J total score, and (B) cumulative duration of hospitalization and MoCA-J total score MoCA-J, Japanese versions of the Montreal Cognitive Assessment

To investigate the effects of age on cognitive function, Pearson's correlation analysis was conducted. It revealed a significant negative correlation between age and MoCA-J total score (ρ = −0.51, p < 0.01) (Figure 3A). No significant correlation was found between the cumulative duration of hospitalization and MoCA-J total score (Figure 3B).

Furthermore, to investigate the influence of age on MCT outcomes, participants in the OT+MCT group were divided into two subgroups based on the median age (68 years): those younger than 68 years (n = 11, 59.82 [SD = 9.62] years) and those 68 years or older (n = 10, 75.30 [SD = 3.68] years). Changes in assessment scores (post-intervention minus pre-intervention) were compared between the two groups (Table 3). There were no significant differences in the changes in the MoCA-J or GAF scores between the two groups. Although the group aged 68 years or younger showed an increase in SR scores on the BCIS after the intervention, and a significant group difference was observed at post-assessment, the within-group change from baseline was not statistically significant. In contrast, the reduction in PANSS scores was greater in participants aged 68 years or older than in the younger group. While no significant difference was observed in positive symptoms (p = 0.11, d = 0.78), significant differences were observed in negative symptoms (p = 0.007, d = 1.77), general psychopathology (p = 0.001, d = 2.07), and total scores (p = 0.001, d = 1.72).

**Table 3:** Comparison of score changes by age at baseline and at four months after the intervention Student’s t-test: *p < 0.05, **p < 0.01 ES, Effect size (*Cohen's d*): *d* = 0.2 (small), *d* = 0.5 (medium), *d* = 0.8 (large). PANSS, Positive and Negative Syndrome Scale; MoCA-J, Japanese version of the Montreal Cognitive Assessment; BCIS, Beck Cognitive Insight Scale; GAF, Global Assessment of Functioning.

Variable		Younger than 68 years (n = 11)	68 years and older (n = 10)	t	p	ES ( d )
		Mean ± SD	Mean ± SD			
PANSS						
･Positive	Baseline	20.36 ± 3.78	20.80 ± 6.82	0.18	0.86	0.08
	Post	20.36 ± 5.92	17.60 ± 6.95	0.98	0.34	0.45
	Change	0.00 ± 4.45	–3.20 ± 4.13	1.70	0.11	0.78
･Negative	Baseline	22.82 ± 6.08	23.20 ± 5.25	0.15	0.88	0.07
	Post	23.00 ± 5.76	19.30 ± 5.33	1.52	0.14	0.70
	Change	0.18 ± 2.14	–3.90 ± 3.88	3.03	0.007**	1.39
･General psychopathology	Baseline	42.82 ± 8.09	48.00 ± 9.91	1.32	0.20	0.61
	Post	42.36 ± 10.55	37.90 ± 8.15	1.05	0.31	0.48
	Change	–0.46 ± 4.70	–10.10 ± 6.66	3.87	0.001**	2.07
･Total score	Baseline	86.00 ± 14.84	92.00 ± 19.57	0.80	0.44	0.37
	Post	85.73 ± 19.76	74.80 ± 19.17	1.23	0.21	0.59
	Change	–2.30 ± 9.21	–17.20 ± 11.49	3.74	0.001**	1.72
MoCA-J						
･Visuospatial/Executive	Baseline	2.00 ± 1.41	1.90 ± 1.45	0.16	0.87	0.07
	Post	2.55 ± 1.51	2.50 ± 1.65	0.07	0.95	0.03
	Change	0.55 ± 1.13	0.60 ± 1.08	0.11	0.91	0.05
･Naming	Baseline	2.73 ± 0.65	2.70 ± 0.48	0.11	0.91	0.05
	Post	2.73 ± 0.47	2.60 ± 0.70	0.49	0.63	0.23
	Change	0.00 ± 0.45	–0.10 ± 0.57	0.45	0.66	0.21
･Attention	Baseline	3.91 ± 1.70	3.50 ± 1.84	0.53	0.60	0.24
	Post	4.73 ± 1.74	4.00 ± 1.63	0.99	0.34	0.45
	Change	0.81 ± 1.25	0.50 ± 1.18	0.60	0.55	0.27
･Language	Baseline	1.46 ± 0.82	1.10 ± 0.99	0.89	0.38	0.41
	Post	1.09 ± 0.70	1.10 ± 0.74	0.03	0.98	0.01
	Change	–0.36 ± 0.80	0.00 ± 0.94	0.95	0.35	0.44
･Abstraction	Baseline	0.91 ± 0.70	0.70 ± 0.82	0.63	0.54	0.29
	Post	0.91 ± 0.94	1.10 ± 0.88	0.48	0.64	0.23
	Change	0.00 ± 0.63	0.40 ± 0.84	1.24	0.23	0.57
･Delayed Recall	Baseline	1.09 ± 1.64	1.30 ± 1.70	0.29	0.78	0.13
	Post	1.18 ± 1.78	1.70 ± 2.00	0.63	0.54	0.29
	Change	0.09 ± 1.04	0.40 ± 1.27	0.61	0.55	0.28
･Orientation	Baseline	5.64 ± 0.67	4.90 ± 1.20	1.76	0.09	0.81
	Post	5.46 ± 1.03	4.80 ± 1.48	1.19	0.25	0.54
	Change	–0.18 ± 1.08	–0.10 ± 1.10	0.17	0.87	0.08
･Total score	Baseline	18.46 ± 5.20	16.80 ± 5.70	0.70	0.50	0.31
	Post	19.36 ± 5.28	18.60 ± 5.32	0.33	0.75	0.15
	Change	0.91 ± 2.34	1.80 ± 1.32	1.06	0.30	0.49
BCIS						
･Self-reflectiveness	Baseline	11.00 ± 4.47	10.00 ± 3.40	0.57	0.57	0.26
	Post	14.08 ± 4.44	9.90 ± 4.58	2.13	0.04*	0.98
	Change	3.09 ± 4.32	–0.10 ± 4.43	1.67	0.11	0.77
･Self-certainty	Baseline	8.46 ± 4.37	7.19 ± 3.26	0.75	0.47	0.34
	Post	9.27 ± 2.61	8.60 ± 4.50	0.42	0.68	0.19
	Change	0.82 ± 3.60	1.41 ± 3.95	0.36	0.72	0.16
･Composite index	Baseline	2.55 ± 4.63	2.83 ± 5.71	0.12	0.90	0.06
	Post	4.82 ± 4.90	1.30 ± 6.80	1.37	0.19	0.63
	Change	2.27 ± 3.61	–1.53 ± 4.70	0.96	0.05	0.96
GAF						
	Baseline	44.36 ± 13.63	48.50 ± 20.77	0.54	0.59	0.25
	Post	46.18 ± 14.25	50.30 ± 22.65	0.50	0.62	0.23
	Change	1.82 ± 4.90	1.80 ± 7.38	0.01	0.99	0.00

## Discussion

This is one of the few studies that examined the effectiveness of MCT in improving psychiatric symptoms in elderly patients with schizophrenia who have been hospitalized for a long time in Japan [16]. At baseline, the MoCA-J total score was significantly lower than the cutoff value for MCI (26/25) in both groups. Using the Japanese version of the BACS as an external criterion, the severity of MoCA-J was classified as mild impairment (borderline range 21.3; SD = 3.6), mild impairment (18.8; SD = 3.8), and moderate impairment or worse (16.3; SD = 4.3) [27], indicating that most participants had cognitive impairment at the moderate impairment level or worse. Furthermore, baseline PANSS and GAF scores indicated severe psychiatric symptoms and functional impairments in both groups. These baseline findings are consistent with previous reports [4-7]. Establishing treatment strategies to improve cognitive function and psychiatric symptoms is an important challenge in Japanese psychiatric hospitals, where many patients are hospitalized long-term.

In the OT+MCT group, no participant discontinued the intervention, confirming the feasibility of introducing MCT for elderly patients with schizophrenia. The MCT sessions were conducted in closed groups composed of long-term hospitalized participants who were familiar with one another; consequently, they appeared comfortable and engaged from the start, with minimal signs of anxiety. Furthermore, participants showed interest in the photos and animations included in modules such as a jumping-to-conclusions bias (modules 2 and 7), a bias against disconfirmatory evidence (module 3), deficits in theory of mind (modules 4 and 6), and they appeared to enjoy the tasks. This engagement was likely a key factor contributing to the high participation rate.

The increase in MoCA-J visuospatial/executive and total scores observed in the post-intervention evaluation indicates a slight improvement in cognitive function. However, no interaction between within-subjects and between-subjects factors was observed, making it difficult to attribute these improvements specifically to MCT. In the MoCA-J naming task (in which participants name animals depicted in pictures), an interaction between within-subjects and between-subjects factors was observed. However, this was due to a slight decline in performance in the OT+MCT group in the post-intervention evaluation, and further confirmation through retesting is warranted. A systematic review and meta-analysis conducted by Jeffrey et al. (2025) demonstrated that MCT is ineffective in improving neurocognitive function [28]. The primary goal of MCT is to modify the cognitive underpinnings of delusional beliefs, including a jumping-to-conclusions bias, a bias against disconfirmatory evidence, deficits in theory of mind, overconfidence in memory errors, and depressive cognitive patterns. In a previous MCT study conducted in Japan, an improvement trend in cognitive function (particularly language memory and attention) was observed in hospitalized patients with schizophrenia [16]. Even if improvements in cognitive function are observed following MCT, these may be secondary cognitive improvements associated with changes in delusional beliefs or thought patterns. A negative correlation was found between the total MoCA-J score and the age of the participants, but no clear correlation was observed with the cumulative hospitalization duration. These results suggest that age may have a greater impact on cognitive decline than the length of hospitalization in patients with schizophrenia.

In the post-intervention evaluation, significant reductions were observed in the PANSS general psychopathology and total scores due to within-subjects factors, with a tendency toward greater score reductions in the OT+MCT group than in the OT-alone group. In addition, negative symptoms showed significant differences between the two groups due to between-subjects factors, with a reduction in negative symptoms observed in the OT+MCT group. These results suggest a trend toward improvement in psychiatric symptoms with MCT. Although the efficacy of MCT in improving positive symptoms, particularly delusions, has been reported [18-20], in the present study, changes in positive symptoms on the PANSS were minimal, while improvements in negative symptoms and general psychopathology were observed. These results may suggest limitations in improving positive symptoms in patients with schizophrenia who have been hospitalized for a long time. In addition, when comparing younger (<68 years) and older (≥68 years) participants within the OT+MCT group, significantly greater improvements in PANSS negative symptoms, general psychopathology, and total score were observed in the older group, indicating a stronger therapeutic effect of MCT in older patients. Although the underlying reasons for this age-related difference remain unclear, previous studies have noted that elderly long-term hospitalized patients often experience a decline in social skills and activities of daily living [5,6]. The structured social interaction and cognitive engagement in MCT sessions likely played a role in stabilizing psychiatric symptoms in elderly patients with schizophrenia.

Strengths and limitations

A key strength of this study is its demonstration of the feasibility of incorporating MCT into treatment for patients with schizophrenia undergoing long-term hospitalization. Importantly, MCT was effective in alleviating psychiatric symptoms. These findings provide evidence supporting the application of the MCT in patients with long-term hospitalization and schizophrenia who continue to exhibit psychiatric symptoms.

This study has some limitations. First, the study was conducted at a single facility with a small sample size, which may have caused group imbalances and limited the statistical power of the results. Future studies should include more participants to enhance result reliability. Second, occupational therapy activities, which are treatment elements other than MCT, and the fact that the daily living environment was not controlled. Because many participants lived in the same ward, daily interactions may have led to the sharing of MCT-related content between the OT+MCT and OT-alone groups. Third, the number and frequency of the MCT sessions may have been insufficient for this population. The current format of 16 weekly sessions over four months may not have been adequate for long-term inpatients. Further research is required to determine the appropriate frequency and duration of MCT in patients hospitalized for long periods. The fourth limitation is that the improvement in psychiatric symptoms demonstrated by MCT in this study should be considered only a short-term effect. To determine the long-term therapeutic effects of MCT, a follow-up period should be set and the results reevaluated. Fifth, the effects of different psychotropic drugs were not adequately considered. Long-term administration of benzodiazepines has been associated with impaired attention and working memory in patients with schizophrenia [29], and the dosage of anticholinergic drugs is significantly correlated with cognitive function test results [30]. Future studies should increase the sample size and include analyses that account for the effects of these medications to isolate the specific impact of MCT. 

## Conclusions

Patients with schizophrenia who have been hospitalized for a long time exhibited cognitive impairments of at least moderate severity, along with severe psychiatric symptoms. In the OT+MCT group, MCT was completed without interruption, confirming its feasibility for patients with schizophrenia who have been hospitalized for a long time in psychiatric hospitals. No improvement in cognitive function was observed after MCT implementation. However, the OT+MCT group showed greater improvement in psychiatric symptoms than did the OT-alone group, with a tendency toward greater improvement in the older (≥68) group. The study results provide evidence supporting the application of MCT in long-term hospitalized patients with schizophrenia and residual psychiatric symptoms.
